# Reducing the Nitrate Content in Vegetables Through Joint Regulation of Short-Distance Distribution and Long-Distance Transport

**DOI:** 10.3389/fpls.2020.01079

**Published:** 2020-07-16

**Authors:** Guihong Liang, Zhenhua Zhang

**Affiliations:** College of Resources and Environmental Sciences, Hunan Agricultural University, Changsha, China

**Keywords:** nitrate, proton pumps, chloride channel protein, NPF7.3, NPF7.2, vegetable, health

## Abstract

As an important nitrogen source, nitrate (NO_3_^−^) absorbed by plants is carried throughout the plant *via* short-distance distribution (cytoplasm to vacuole) and long-distance transportation (root to shoot), the two pathways that jointly regulate the content of NO_3_^−^ in plants. NO_3_^−^ accumulation within the vacuole depends on the activities of both tonoplast proton pumps and chloride channel (CLC) proteins, and less NO_3_^−^ is stored in vacuoles when the activities of these proteins are reduced. The ratio of the distribution of NO_3_^−^ in the cytoplasm and vacuole affects the long-distance transport of NO_3_^−^, which is regulated by the proteins NPF7.3 and NPF7.2 that play opposite but complementary roles. NPF7.3 is responsible for loading NO_3_^−^ from the root cytoplasm into the xylem, whereas NPF7.2 regulates the unloading of NO_3_^−^ from the xylem, thereby facilitating the long-distance transport of NO_3_^−^ through the roots to the shoots. Vegetables, valued for their nutrient content, are consumed in large quantities; however, a high content of NO_3_^−^ can detrimentally affect the quality of these plants. NO_3_^−^ that is not assimilated and utilized in plant tissues is converted *via* enzyme-catalyzed reactions to nitrite (NO_2_^−^), which is toxic to plants and harmful to human health. In this review, we describe the mechanisms underlying NO_3_^−^ distribution and transport in plants, a knowledge of which will contribute to breeding leafy vegetables with lower NO_3_^−^ contents and thus be of considerable significance from the perspectives of environmental protection and food safety.

## Introduction

Nitrogen (N) is an essential element that affects the growth, yield, and quality of crops (Peng et al., 2010; Ju et al., 2015). It is required for the synthesis of macromolecular compounds such as proteins and nucleic acids, which play important roles in the metabolism and energy production of organisms (Li and Gong, 2011). Ammonium nitrogen (NH_4_^+^-N) and nitrate nitrogen (NO_3_^−^-N) are the main forms of nitrogen absorbed and utilized by crops, with the latter being the main nitrogen source for dryland crops (Tang et al., 2013; Zhao et al., 2018). Exogenous concentrations of NO_3_^−^ are among the most important factors affecting the accumulation of NO_3_^−^ in plants. Plants actively take up NO_3_^−^ from the environment through a proton/nitrate-coupled mechanism (Wang et al., 2018). Several NPF/peptide transporter (PTR) family and NO_3_^−^ transporter 2 (NRT2) members in *Arabidopsis* are involved in NO_3_^−^ uptake, including components of the high-affinity transport system [HATS: NRT2.1, NRT2.2 (Filleur et al., 2001), NRT2.4 (Kiba et al., 2012), and NRT2.5 (Lezhneva et al., 2014)] and low-affinity transport system [LATS: NPF4.6 (Huang et al., 1999)]. Furthermore, NPF6.3 is known to function as a dual-affinity NO_3_^−^ transporter (Liu et al., 1999), the affinity for NO_3_^−^ of which is determined by external NO_3_^−^ concentrations (Ho et al., 2009). Previous studies have shown that differences in NO_3_^−^ accumulation in different lettuce varieties are largely attributable to differences in NO_3_^−^ uptake (Burns et al., 2011). In a study examining two varieties of Chinese cabbage with high and low NO_3_^−^ accumulation, respectively, a stronger NO_3_^−^ absorption capacity and higher expression of *NPF* and *NRT2* genes were detected in the high NO_3_^−^-accumulating variety (Zhao et al., 2011). The NO_3_^−^ absorbed by plants can be directly assimilated into amino acids by the activities of NO_3_^−^ reductase (NR), nitrite reductase (NiR), glutamine synthetase (GS), and glutamate synthase (GOGAT) in the cell cytosol (Williams et al., 1987). An imbalance in the efficiency of NO_3_^−^ absorption and reduction in vegetables has been demonstrated to be an important factor in determining NO_3_^−^ accumulation, and the activity of NR has been shown to be significantly negatively correlated with the NO_3_^−^ content in Chinese cabbage leaves (Du et al., 2008). Moreover, a proportion of NO_3_^−^ is secreted into the rhizosphere, mediated by the NO_3_^−^ transporter NPF2.7 (Wang et al., 2018).

The NO_3_^−^ that remains unutilized by plants is distributed throughout the plant *via* short-distance distribution and long-distance transportation (Han et al., 2015; Zhang, 2017). Short-distance distribution of NO_3_^−^ occurs at the subcellular level and involves the distribution of NO_3_^−^ between the cytoplasm and vacuoles, a process regulated by members of the chloride channel (CLC) protein family. Initially, it was believed that CLC proteins were involved only in the transport of Cl^−^, functioning as chloride channels and Cl^−^/H^+^ anti-transporters. However, in a study examining the *Arabidopsis clca* mutant, De Angeli et al. (2006) demonstrated that CLC family proteins could also transport NO_3_^−^ across the tonoplast by acting as 2NO_3_^−^/H^+^ antiporters. Compared with wild-type plants, the *clca* mutant was found to accumulate 50% less NO_3_^−^ in the vacuole, thereby indicating that *AtCLCa* plays a key role in regulating the vacuolar storage and short-distance distribution of NO_3_^−^ in plants (De Angeli et al., 2006).

The long-distance transport of NO_3_^−^ mainly relies on the coordination of the NPF proteins with low affinity. AtNPF7.3 is a pH-dependent NO_3_^−^ transporter protein located in the cytomembrane and expressed in the pericycle cells near the xylem. It has been found that in mutants lacking *AtNPF7.3*, the content of NO_3_^−^ in the xylem sap and shoots is significantly reduced, thereby indicating that *AtNPF7.3* plays an important role in promoting the long-distance transport of NO_3_^−^ from roots to aerial parts (Lin et al., 2008). In contrast, AtNPF7.2 is mainly expressed in parenchyma cells near the root xylem, where it functions in the unloading of NO_3_^−^ from the xylem to regulate the distribution ratio of NO_3_^−^ between the roots and shoots (Li et al., 2010). The concentration of NO_3_^−^ in plants is thus affected by multiple processes, among which there is a close interdependency that facilitates the coordinated regulation of NO_3_^−^ accumulation in plants.

As an important source of inorganic nitrogen, the NO_3_^−^ content of food products may originate from soil, water resources, chemical fertilizers, or food additives, consequently affecting the human food chain (Quijano et al., 2017). To date, numerous studies have been conducted on the NO_3_^−^ content of plants and have revealed high concentrations of NO_3_^−^ in different vegetables (Bahadoran et al., 2016), particularly leafy vegetables, which are the most important source of NO_3_^−^ in the human diet, accounting for more than 80% of the NO_3_^−^ intake (Anjana and Iqbal, 2007; Bondonno et al., 2018; Chetty et al., 2018). However, during the storage and processing of plants, excess NO_3_^−^ that remains unassimilated in plant tissues can be enzymatically converted to NO_2_^−^. The NO_3_^−^ ingested by humans can also be reduced to NO_2_^−^ *via* the activity of gut microorganisms. NO_2_^−^ is a strong carcinogen and causes the accumulation of methemoglobin, a compound with potentially toxic effects on human health (Salehzadeh et al., 2020). The content of NO_3_^−^ in vegetables depends on its distribution and utilization and has become established as one of the indices used to assess the quality of vegetables and their processed products. Consequently, measures that can be used to reduce the concentration of NO_3_^−^ in leafy vegetables, by regulating its distribution and utilization, are of importance with respect to reducing environmental pollution and ensuring a healthy diet.

## Short-Distance (Vacuole to Cytoplasm) NO_3_^−^ Distribution in Vegetables

The evolutionary success of higher plants is largely attributable to their unique cellular architecture (Kriegel et al., 2015). Vacuoles, as the largest organelles in mature plant cells, account for approximately 90% of the cell volume and are generally utilized for the storage of nutrients (Liao et al., 2002; Shen et al., 2003; Krebs et al., 2010; Liao et al., 2018). Having been absorbed by plants, NO_3_^−^, an important plant nutrient, is allocated to the metabolic pool (cytoplasm) or the storage pool (vacuole) (Miller et al., 2007). The concentrations of NO_3_^−^ in the vacuole and cytoplasm are typically within the ranges of 30 to 50 mol m^−3^ and 3 to 5 mol m^−3^, respectively (Martinoia et al., 2000), and the fraction stored in the vacuole cannot be metabolized by plants unless it is initially allocated to the surrounding cytoplasm. The vacuole is accordingly the main storage site of NO_3_^−^, and thus the role of this vacuolar fraction in NO_3_^−^ distribution should not be overlooked in studies on plant NO_3_^−^ contents (Martinoia et al., 1981; Huang et al., 2012; Han et al., 2015).

**Figure 1:**
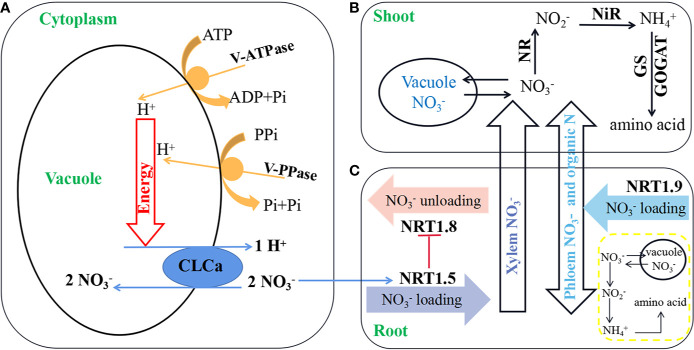
A model of short-distance distribution and long-distance transport of NO_3_^−^ in plants. Modified from Zhang (2017). **(A)** Accumulation of NO_3_^−^ in the vacuole is regulated by chloride channels (CLCa) and relies on an H^+^ gradient established by V-ATPase and V-PPase. **(B)** Short-distance distribution of NO_3_^−^ and reactions catalyzed by NO_3_^−^ reductase (NR), nitrite reductase (NiR), glutamine synthetase (GS), and glutamate synthase (GOGAT) in shoots. **(C)** Long-distance transport of NO_3_^−^ from roots to shoots is mainly regulated by *NPF7.3*, *NPF7.2*, and *NPF2.9* genes.

In general, the assimilatory power of leaf cell cytoplasm is sufficient with respect to reducing NO_3_^−^ concentrations. As long as NO_3_^−^ remains distributed outside the vacuole, it can be rapidly assimilated, and therefore the content of NO_3_^−^ in plants is largely determined by the ratio of the distribution of NO_3_^−^ within and outside the vacuole (Shen et al., 2003; Huang et al., 2012; Han et al., 2016). Changes in the relative size of these two NO_3_^−^ reservoirs are mediated by the 2NO_3_^−^/H^+^ exchange channel CLCa in the tonoplast (Krebs et al., 2010; Liao et al., 2019) and is dependent on the activity of the vacuolar H^+^-ATPase (V-ATPase) and the vacuolar H^+^-pyrophosphatase (V-PPase) proton pumps. The combined activity of these vacuolar proton pumps, utilizing MgATP and MgPPi as substrates, respectively, establishes an electrochemical H^+^ gradient across the tonoplast that drives the transport of NO_3_^−^ against its electrochemical potential (Martinoia et al., 2007; Lin et al., 2012) (**Figure 1A**).

These two proton pumps are among the most abundant tonoplast proteins, thereby indicating the considerable amounts of energy expended in vacuolar transport (Carter et al., 2004; Jaquinod et al., 2007). V-ATPases are highly conserved, multi-subunit proton pumps that consist of two sub-complexes, the peripheral V_1_ and membrane-integral V_0_ complexes. The peripheral V_1_ complex comprises eight subunits (VHA-A to -H), which are exposed on the cytoplasmic side of the vacuolar membrane and are responsible for ATP hydrolysis. The membrane-integral V_0_ complex, which consists of six subunits (VHA-a, -c, -cʹ, cʹʹ, -d, and -e), is integrated within the membrane and functions as a channel for the translocation of protons from the cytoplasm into the lumen of endomembrane compartments and also serves as the binding site for the polymerization and assembly of V_1_ subunits (Cipriano et al., 2008; Luo, 2012). Compared with tonoplast H^+^-ATPase, the H^+^-PPase proton pump, which is widely distributed in plants, a few algae, protozoa, and bacteria, has a simple structure comprising an oligopeptide chain and accounts for approximately 1 to 10% of membrane proteins (Zhao and Liu, 1999; Maeshima, 2000).

V-ATPase and V-PPase not only serve as key tonoplast proton pump enzymes but also function as determinants of NO_3_^−^ re-utilization (Shen et al., 2003). From the perspective of reducing NO_3_^−^ accumulation in plant vacuoles and further improving the nitrogen-utilization efficiency of crops, it is of considerable significance to elucidate the short-distance distribution mechanism of NO_3_^−^ associated with tonoplast proton pump activity (Han et al., 2014). In a study examining two varieties of rapeseed seedlings with high and low nitrogen-use efficiency, respectively, Han et al. (2015) revealed that V-ATPase and V-PPase activities and NO_3_^−^ internal flow rate into the vacuole in the highly efficient variety were lower compared with those in the variety with low nitrogen-use efficiency. This indicates that in the former, larger amounts of NO_3_^−^ are assigned to the cytoplasm for subsequent metabolism, thereby reducing the vacuolar content of NO_3_^−^ and consequently enhancing the nitrogen-use efficiency of rapeseed (Han et al., 2016). The findings of a further study have indicated that the storage capacity for NO_3_^−^ is limited in the absence of the *AtVHA-a2* and *AtVHA-a3* genes, with the *vha-a2 vha-a3* double mutant being found to contain 80% less NO_3_^−^, whereas the total NO_3_^−^ reductase activity had increased by 90% (Krebs et al., 2010). Similarly, it has been observed that the vacuoles of *atvha-a2*, *atvha-a3*, and *atavp1* mutants contain less NO_3_^−^ (Liao et al., 2019).

Driven by vacuolar proton pumps, NO_3_^−^ enters the vacuole *via* chloride channel proteins (Gaxiola et al., 2001; De Angeli et al., 2006; Brüx et al., 2008). Chloride and malic acid channels, two main types of anion channels, are inward rectifying channels located in the tonoplast (Qu and Yu, 2008). CLC proteins facilitate the flux of anions such as Cl^−^ and NO_3_^−^, whereas glutamate transport is limited. CLC proteins are activated only in the presence of cytoplasmic Ca^2+^ (Berechi et al., 1999). This activation is promoted by calmodulin domain protein kinase (CDPK) in the presence of ATP and inhibited by niflumic acid. Unlike general anion channels, CLCs are not inhibited by 4,4′-dinitrostilbene-2,2′-disulfonic acid (White and Broadley, 2001; Yu and Liu, 2004; Qu et al., 2009).

In *Arabidopsis*, NRT2.7 and CLCa are two transporters that facilitate the transfer of NO_3_^−^ into the vacuole (Geelen et al., 2000; Chopin et al., 2007). Other transporters with potentially similar functions include CLCc and NPF6.2 (Chiu et al., 2004; Harada et al., 2004). Seven CLC family members have been identified in *Arabidopsis thaliana*, and De Angeli and colleagues (2006) were the first to demonstrate that AtCLCa is a tonoplast-localized 2NO_3_^−^/H^+^ antiporter involved in the regulation of NO_3_^−^ sequestration into vacuoles. It has been found that the accumulation of NO_3_^−^ in the vacuole is reduced by approximately 50% and that the contents of NO_3_^−^ in both shoots and roots are reduced in *atclca* mutant plants (Geelen et al., 2000; De Angeli et al., 2006; Monachello et al., 2009). Similar results were obtained in a study by Liao et al. (2019). AtCLCc is also assumed to participate in vacuolar NO_3_^−^ storage, given that it is tonoplast localized, and plants with a mutation in this proton are characterized by lower NO_3_^−^ contents (Harada et al., 2004). Among the NPF proteins in *Arabidopsis*, AtNPF6.2 is specifically expressed in NO_3_^−^-rich petioles, thereby indicating that it might participate in the accumulation of NO_3_^−^ in petiole cell vacuoles. However, the function of AtNPF6.2 has yet to be conclusively established owing to a lack of evidence to indicate whether this protein is located in the vacuolar membrane (Chiu et al., 2004). AtNRT2.7, which is located in the tonoplast and expressed exclusively in seeds, is, to date, the only protein in the NRT family confirmed to participate in the vacuolar storage of NO_3_^−^. Within seeds, this protein is responsible for the transport of NO_3_^−^ from the cytoplasm into the vacuole for storage, thereby contributing to seed nitrogen accumulation (Chopin et al., 2007). Compared with NPF and NRT2 family proteins, CLC proteins are primarily responsible for vacuolar NO_3_^−^ accumulation during plant growth, and a reduction in their activity enhances the utilization of NO_3_^−^, thereby improving the nitrogen-use efficiency of crops (Geelen et al., 2000; De Angeli et al., 2006; Monachello et al., 2009).

With respect to the efflux of NO_3_^−^ from vacuoles, it has been established that the tonoplast-localized proteins AtCLCb and OsNPF7.2 could transport NO_3_^−^, although there is currently no direct evidence to indicate whether these proteins are involved in vacuolar NO_3_^−^ influx or efflux (von der Fecht-Bartenbach et al., 2010; Hu et al., 2016). AtNPF5.11, AtNPF5.12, and AtNPF5.16, regulate NO_3_^−^ re-allocation between roots and shoots by mediating NO_3_^−^ efflux from the vacuole to the cytosol. In triple mutants characterized by disruption of *AtNPF5.11*, *AtNPF5.12*, and *AtNPF5.16*, it has been found that larger amounts of root-fed ^15^NO_3_^−^ are translocated to the shoots (He et al., 2017). Moreover, it is speculated that the tonoplast-localized OsNPF7.2 could also be involved in NO_3_^−^ efflux from the vacuoles, and heterologous expression in *Xenopus laevis* oocytes has indicated that it mediates NO_3_^−^ absorption, although, to date, it has yet to be demonstrated whether functional disruption of *OsNPF7.2* leads to an accumulation of NO_3_^−^ within the vacuoles (von der Fecht-Bartenbach et al., 2010; Hu et al., 2016). Furthermore, NO_3_^−^ supply has a significant effect on NO_3_^−^ distribution, both in the metabolic pool and the storage pool of leaf blades in three leafy vegetables (rapeseed, Chinese cabbage, and spinach) (Chen et al., 2004).

The 2NO_3_^−^/H^+^ antiporter located in the tonoplast assists in the transfer of NO_3_^−^ into the vacuole from the cytoplasm (De Angeli et al., 2006; Krebs et al., 2010). From the perspective of nitrogen-use efficiency, the role of vacuolar NO_3_^−^ in maintaining an osmotic balance within the vacuole is considered wasteful (Shen et al., 2003). However, whether it would be feasible to replace vacuolar NO_3_^−^ with Cl^−^ to maintain cell osmotic pressure within a certain range, and thereby reduce the level of NO_3_^−^ accumulated in the vacuole, remains to be ascertained.

## Long-Distance (Root to Shoot) No_3_^−^ Transport in Vegetables

A proportion of the NO_3_^−^ absorbed by plant roots is stored within vacuoles or assimilated into organic nitrogen through a series of reactions catalyzed by NO_3_^−^ reductase, nitrite reductase, glutamine synthetase, and glutamate synthase (Williams et al., 1987) (**Figure 1C**). However, most of the NO_3_^−^ taken up by roots is transported through cortical tissues into xylem conduits for long-distance transport to different shoot tissues and organs for subsequent assimilation and utilization in plant growth and development (Han et al., 2016; Zhang, 2017) (**Figure 1B**).

NO_3_^−^ is primarily transported upward by the xylem and downward by the phloem (Dechorgnat et al., 2011). The long-distance transport and distribution of NO_3_^−^ between roots and shoots in *Arabidopsis* are mainly co-regulated by *NPF7.3* and *NPF7.2*, two members of the PTR family, the expression of which is strongly induced by NO_3_^−^ (Lin et al., 2008; Li et al., 2010; Chen et al., 2012; Léran et al., 2014; Zhang Z. H. et al., 2014) (**Figure 1C**). *AtNPF7.3* is predominantly expressed in the columnar sheath cells surrounding the protoxylem in the root and is responsible for the loading of NO_3_^−^ from the root cytoplasm into the xylem for transport to the shoots (Lin et al., 2008). *AtNPF7.2* is expressed in xylem parenchyma cells and affects NO_3_^−^ transport from roots to shoots by regulating NO_3_^−^ unloading from the xylem (Li et al., 2010). Thus, although playing opposing roles in the loading and unloading of NO_3_^−^ into and out of the xylem, *AtNPF7.3* and *AtNPF7.2*, respectively, cooperatively contribute to the long-distance transport of NO_3_^−^ from the roots to the shoots. Furthermore, OsNPF2.2 has been shown to be involved in unloading NO_3_^−^ from the xylem, thereby facilitating root to shoot NO_3_^−^ transport and plant development. *osnpf2.2* mutant plants have been found to maintain high levels of NO_3_^−^ in the roots and have a low shoot:root NO_3_^−^ ratio (Li et al., 2015). Moreover, it has been observed that AtNPF2.3 plays an important role in root to shoot NO_3_^−^ translocation in plants subjected to saline conditions (Taochy et al., 2015).

Currently, *AtNPF2.9* is the only gene that has been confirmed to play a role in the loading of NO_3_^−^ into root phloem and negatively regulates the root to shoot transport of NO_3_^−^ (Fan et al., 2009; Wang and Tsay, 2011). It has also been established that *AtNPF5.11*, *AtNPF5.12*, and *AtNPF5.16* play roles in the uptake of NO_3_^−^ from the vacuole to the cytoplasm in *Arabidopsis* to regulate the shoot:root ratio, and in a triple mutant for these genes, larger amounts of NO_3_^−^ were found to be translocated to the shoots (He et al., 2017). In *Arabidopsis*, AtNPF2.9, AtNPF7.2, and AtNPF7.3 are the main NO_3_^−^ transporters contributing to the regulation of the long-distance transport and redistribution of NO_3_^−^ from roots to shoots (**Figure 1C**). In this regard, it is worth noting that the concentration of NO_3_^−^ transported in the xylem is typically several tens to hundreds of times higher than that carried in the phloem, which accordingly explains why studies on NO_3_^−^ distribution in shoots and roots have focused primarily on NO_3_^−^ transport in the xylem rather than in the phloem (Lin et al., 2008; Li et al., 2010; Wang and Tsay, 2011). To date, research on the relationships between NPF genes and NO_3_^−^ long-distance transport in crop plants has mainly been conducted on wheat, rice, and rapeseed. However, only the responses of the *NPF7.3* and *NPF7.2* genes to nitrogen stress have been studied in wheat (Xuan et al., 2014), whereas the physiological characteristics of nitrogen have been analyzed in rice lines overexpressing OsNPF7.3 (Ma, 2011). In our studies, we have analyzed the response of *NPF7.3* and *NPF7.2* to NO_3_^−^ deficiency and constructed a co-expression network to identify key genes involved in NO_3_^−^ transport from root to shoot in rapeseed seedlings (Hua et al., 2018; Liang et al., 2019). Evidence that has accumulated to date essentially confirms that *NPF7.3* and *NPF7.2* regulate NO_3_^−^ transport and distribution in a range of crop plants, in which they play roles similar to those observed in *A. thaliana*. We thus believe that the relationships between NO_3_^−^ long-distance transport and NPF genes described in *Arabidopsis* would also be applicable to cultivated crops or vegetables.

Xylem flow is known to be strongly influenced by transpiration, which in turn has a considerable effect on the long-distance transport of NO_3_^−^. NO_3_^−^ loaded into the xylem is continuously transported to the above-ground parts of plants, mediated through the influence of the transpirational pull, thereby promoting the transport of NO_3_^−^ from the roots to shoots (Li et al., 2010; Chen et al., 2012). Consistently, it has been demonstrated that the inhibition of transpiration reduces the transport of NO_3_^−^ to the shoots in pea plants, thus resulting in an increase in the accumulation of NO^−^ in the roots and a reduction in contents in the edible parts of plants (Hernandez et al., 1997). The long-distance transport of NO_3_^−^ not only determines the distribution and assimilation in different tissues, but also represents an important physiological mechanism whereby plants respond to environmental change (Zhang G. B. et al., 2014). Chen et al. (2012) and Li et al. (2010) have shown that transpiration regulates the transport of nutrient elements from roots to shoots under normal conditions, whereas NO_3_^−^ accumulation in roots, which is also mediated by *AtNPF7.3* and *AtNPF7.2*, might be the dominant regulatory factor in NO_3_^−^ distribution in plants exposed to stress. For example, under conditions of cadmium stress, wild-type *Arabidopsis* plants have been observed to maintain a higher root to shoot NO_3_^−^ ratio than *atnpf7.2* mutant plants, indicating that this ratio is actively regulated by NPF7.2 and other transporters, rather than passively by transpiration (Li et al., 2010). Therefore, by inhibiting the expression of the *NPF7.3* gene and enhancing that of *NPF7.2* in plants, it might be possible to control the long-distance transport of NO_3_^−^ from roots to shoots, which would represent a useful approach for reducing the content of NO_3_^−^ in leafy vegetables.

## Conclusions and Future Developments

In plants, NO_3_^−^ accumulation depends on its absorption and metabolism. Plants actively take up NO_3_^−^ from the environment through a proton/nitrate-coupled mechanism (Wang et al., 2018). The NO_3_^−^ absorbed by plants can be directly assimilated into organic nitrogen, secreted into the rhizosphere, accumulated in the vacuole, or transported from roots to shoots (Zhang, 2017). The concentrations of NO_3_^−^ in plants are thus affected by multiple processes, among which there is a close interdependency that facilitates the coordinated regulation of NO_3_^−^ accumulation in plants.

Vacuolar NO_3_^−^ accumulation and release play important roles in regulating the concentration and re-allocation of NO_3_^−^. The influx of vacuolar NO_3_^−^ is mainly affected by the tonoplast-localized proteins, such as AtCLCa, AtCLCc, AtNPF6.2, and AtNRT2.7, whereas AtCLCb and OsNPF7.2 are believed to play transporting roles, although there is currently no direct evidence to indicate whether these proteins are involved in vacuolar NO_3_^−^ influx or efflux (von der Fecht-Bartenbach et al., 2010; Hu et al., 2016). Furthermore, AtNPF5.11, AtNPF5.12, and AtNPF5.16 regulate NO_3_^−^ re-allocation between roots and shoots by mediating NO_3_^−^ efflux from the vacuole to the cytosol (He et al., 2017). However, the main factors regulating the short-distance of NO_3_^−^ are the tonoplast CLC proteins and proton pump transport systems (Chiu et al., 2004; Chopin et al., 2007). The NO_3_^−^ transport system is present in the vacuolar membrane in all plant tissues and represents the main process whereby short-distance NO_3_^−^ distribution is regulated (De Angeli et al., 2006; Krebs et al., 2010). By inhibiting the activity of both tonoplast proton pumps and the CLC proteins, a larger proportion of plant NO_3_^−^ could be re-distributed in the cytoplasm, wherein it can subsequently be metabolized, thereby contributing to a reduction in the NO_3_^−^ content of vegetables.

The vacuolar–cytoplasmic distribution of NO_3_^−^ affects not only the distribution and utilization of NO_3_^−^ (Gaxiola et al., 2001; Brüx et al., 2008; Krebs et al., 2010), but also affects the expression and function of genes induced by NO_3_^−^ (Glass et al., 2002; Huang et al., 2013). *NPF2.9*, *NPF7.3*, and *NPF7.2*, which are responsible for NO_3_^−^ loading in the phloem, loading in the xylem, and unloading from the xylem, respectively, are the main regulators of long-distance NO_3_^−^ transport. However, the phloem is primarily involved in the transport of organic nitrogen, whereas the concentration of inorganic nitrogen carried in the phloem is typically in the order of tens to hundreds of times smaller than that in the xylem (Lin et al., 2008; Fan et al., 2009; Li et al., 2010; Wang and Tsay, 2011). Therefore, NPF7.3 and NPF7.2 are the main regulators of the long-distance transport of NO_3_^−^. Enhancing the activity of the NPF7.2 protein in conjunction with a reduction in the activity of the NPF7.3 protein would contribute to favorably regulating the long-distance transport of NO_3_^−^, thus reducing the transport of NO_3_^−^ from roots to shoots, and thereby the contents of NO_3_^−^ in leafy vegetables.

Research on mechanisms underlying the short- and long-distance translocation of NO_3_^−^ needs to be further expanded to address the following questions (Li et al., 2010; Schroeder et al., 2013). (1) In addition to regulating osmotic functions, how does the storage of NO_3_^−^ in vacuoles affect the expression and function of its inducible genes? (2) What is the difference between the contribution of chloride channels (CLC) and proton pumps with respect to the distribution of NO_3_^−^ in vacuoles? (3) Given that the transport of NO_3_^−^ from cytoplasm to vacuoles is primarily regulated by CLCa, how does this process affect the further assimilation of NO_3_^−^? On the basis of the findings of previous studies, the vacuolar NO_3_^−^ distribution system, which plays an important role in the short-distance NO_3_^−^ distribution, and the vascular bundle long-distance transportation system, which is important in long-distance NO_3_^−^ transportation, are identified as primary targets to further studies on mechanisms underlying the short-distance distribution and long-distance transport of NO_3_^−^. Gaining a better understanding of these mechanisms will contribute to facilitating a more effective control of NO_3_^−^ contents in plants and provide important guidance for the breeding and cultivation of leafy vegetables with low NO_3_^−^ concentrations.

## Author Contributions

GL organized and wrote the original manuscript. GL and ZZ discussed and revised the manuscript and approved the final version.

## Funding

This work was financially supported in part by the National Key R&D Program of China (2017YFD0200100 and 2017YFD0200103), the Hunan Provincial Recruitment Program of Foreign Experts, the National Oilseed Rape Production Technology System of China, “2011 Plan” supported by The Chinese Ministry of Education, and the Double FirstClass Construction Project of Hunan Agricultural University (kxk201801005).

## Conflict of Interest

The authors declare that the research was conducted in the absence of any commercial or financial relationships that could be construed as a potential conflict of interest.
